# Family planning knowledge, use, and associated factors among women with mental illness and epilepsy in Rwanda: a cross-sectional study

**DOI:** 10.3389/fgwh.2024.1373051

**Published:** 2024-10-25

**Authors:** Pacifique Mukangabire, M. Providence Umuziga, Benoite Umubyeyi, Donatilla Mukamana, Darius Gishoma, Vedaste Baziga, Philomene Uwimana, Fidele Sebera, Olive Bazirete, Clementine Kanazayire

**Affiliations:** ^1^ School of Nursing and Midwifery, University of Rwanda, Kigali, Rwanda; ^2^ Department de Recherche, Enseignement et Formation, Maison Medicale Jeanne Garnier, Paris, France; ^3^ Ndera Neuro-Psychiatric Hospital, Kigali, Rwanda

**Keywords:** family planning, women, mental illness, epilepsy, psychiatric disorders

## Abstract

**Introduction:**

Family planning knowledge and access to quality family planning services occupy a central position in the lives of all women of reproductive age. However, women with mental illness and epilepsy who are at a high risk of sexual violence, unwanted pregnancies, poor obstetric outcomes, and drug interaction consequences, need it the most. Understanding their family planning knowledge and utilization remains crucial for meeting their needs. The present study aims to assess knowledge, use of family planning, and associated factors among women living with mental illness and epilepsy who attend the Ndera Neuropsychiatric Hospital and affiliated branches.

**Methodology:**

A cross-sectional study was conducted between October 2022 and February 2023. The study involved a purposive sample of 289 women who attended the Ndera Neuropsychiatric Hospital and its two affiliated branches during the data collection period. Psychiatric nurses administered a structured questionnaire. Data were analyzed using descriptive statistics, and multiple logistic regression analysis was performed to assess the factors associated with the use of family planning methods.

**Results:**

Out of 289 who participated in the study, the majority (96.9%) were aware of family planning methods, most (67.8%) had used one method once in their life course, a half (51.9%) were using a family planning method at the time of data collection, and a slight number (26%) expressed intentions of using a family planning method in the future. The most known and used methods were respectively the injectable (17.5%) and oral contraceptive pill (17.5%). Regarding the natural family planning methods, breastfeeding and withdrawal were less used. Being single (AOR = 66.4, 95% CI: 9.8, 44) or married (AOR = 51.4, 95% CI: 11.9–22), having a primary level of education (AOR = 5.6, 95% CI: 2.0, 15.9), knowing a contraceptive method (AOR = 5.8, 95% CI: 0.6, 49) and suffering from brief psychotic disorders (AOR = 2.7, 95% CI: 1.1, 6.6) predicted a higher utilization of family planning.

**Conclusion:**

Most of the women with mental illness in this study were aware of family planning methods and had used one of the family planning methods in life. The national average is below when it comes to family planning awareness. It is important to improve family planning education and counseling for women who attend psychiatric outpatient clinics.

## 1 Introduction

The World Health Organization strives to include people with mental illnesses in society (1). Motherhood and other reproductive health-related issues require specific attention among women with mental illness. Family planning is important for all women of reproductive age, but women with mental diseases, such as schizophrenia, bipolar disorder, and severe depression, may face additional problems and concerns (2). Family planning (FP) is particularly important for women with mental illness since they carry a high risk of sexual violence, unwanted pregnancies, and poor obstetric outcomes due to their mental illness (3). A retrospective study from the Epilepsy Birth Control Registry web-based survey of women with epilepsy (WWE) reported that 65% of pregnancies in women with epilepsy were unplanned, more common among younger, and 34.7% of unintended pregnancies occurred in women with epilepsy who were not taking contraceptives (4). Nevertheless, a study conducted in Japan found that WWE with planned pregnancies were less likely to have seizures during pregnancy and less likely to undergo medication changes (5). It is, therefore, very crucial for women with epilepsy to have access to reproductive health services including FP methods to lower the risks that may be associated with maternal epilepsy.

Despite the increased sexual and reproductive health risks associated with mental illness and epilepsy, access to, and utilization of FP by women with mental health and epilepsy remains low globally, particularly within resources-limited countries (3). Factors previously reported to affect the use of family planning included the influence of severe mental health illness symptoms, knowledge and attitudes regarding family planning, stigma, reproductive coercion, and sexual violence (6). Additionally, compared to other women without mental health conditions, those with mental illness and epilepsy demonstrated a lack of awareness, less reproductive and contraceptive knowledge, and less use of birth control methods with no mental illness history (3, 7, 8).

In Rwanda, the recent mental health survey involving the general population revealed that females are more affected by mental illness compared to males, with a proportion of 23.2% and 16.6% respectively (9). Researchers investigated mental-health-seeking behaviors in Rwandan men and women and its social and gender implications. It was found that both men and women with mental health issues were unable to fully benefit from mental health services available due to the considerable influence of stigma and persisting traditional roles in some mental healthcare settings (10). Similarly, lack of FP awareness and cultural misbelieves about FP are among the factors that hinder FP utilization in the general population (11). It is hypothetical whether women with mental health illnesses seek reproductive health services, specifically family planning, and little is known about factors that might hinder or support their FP-seeking behaviors.

Mental health services in Rwanda are provided through community, district, and referral hospital levels. One of the referral hospitals providing mental health and neurological services is the Ndera Neuropsychiatric Hospital, with two affiliated branches. Rwanda Health Sector report confirmed that 73,675 mental health cases were followed up by Caraes Ndera hospital and its branches, of which 69.831% were treated at CARAES Ndera, 20.661% at CARAES Butare, and 9.507% at Centre Icyizere Kicukiro (12). To the authors' knowledge, no family planning services are provided in these settings, and little is known about the knowledge and utilization of FP methods among women consulting these centers. This study aimed to assess the knowledge, utilization, and factors that might influence the use of FP by women with mental illness and epilepsy in Rwanda.

## 2 Methods

### 2.1 Research design and setting

A cross-sectional descriptive study was done to explore the FP knowledge, use, and associated factors among women with mental illness and epilepsy in Rwanda. The study site was the Ndera Neuropsychiatric Hospital and its branches. The hospital is a referral neuropsychiatric hospital located in Kigali and provides multidisciplinary inpatient and outpatient mental health and neurological services. It has two affiliated branches, one located in Southern Province, Huye District, known as CARAES-Butare, and another one located in Kigali City, Kicukiro District, known as Icyizere. Both branches provide mental health services.

### 2.2 Study population

The study included all women of reproductive age, who are living with mental illness and epilepsy with a stable mental health status and recommended by the physicians in charge who have been following them for at least 6 months.

### 2.3 Sample size

G*Power software as a free power analysis program for a variety of statistical tests commonly used in the social, behavioral, and biomedical sciences (13) was used to determine the sample size. With an effect size of 0.05, α error probability = and 0.05, Power (1-β error probability) = 0.95, the approximated sample size calculated was 396 women attending the Ndera Neuropsychiatric Hospital and its branches. Although, the study collected data from 289 women who attended those facilities during the period of data collection.

### 2.4 Sampling procedure

All women of reproductive age (15–49) who have an individual history of mental illness or epilepsy, who had a stable mental health status, and who attended the three centers during the study period were eligible to participate in the study.

Through a purposive sample procedure, we selected women whose current stable mental health status was confirmed by their attending physician and were scheduled to be discharged from Ndera Hospital, CARAES Butare, and Icyizere centers, and those who were visiting the outpatient services of these settings as follow-up visits after discharge between October 2022 and February 2023.

Women whose mental status was judged not compatible with their participation in a study were excluded. This age category was based on the recommended WHO reproductive age limit, as well as the Rwanda DHS (14). These criteria were set to ensure that the mental status of potential participants is stabilized enough to allow them to articulate more coherently.

### 2.5 Data collection methods

A structured questionnaire developed by Prachi et al. for assessing knowledge, attitude, and practice of family planning among the women of reproductive age group was adopted and adopted in the context of the study (15). The questionnaire included the following sections: socio-demographic characteristics, clinical mental health characteristics of the participants, reproductive characteristics of the respondents, fertility intentions, family planning awareness and methods used, self-reported source of information about contraception, and factors associated with family planning utilization. The questionnaire was translated into the local language “Kinyarwanda” spoken by participants and back-translated to English. The questionnaire was administered by experienced psychiatric nurses working at Ndera Neuropsychiatric Hospital, Caraes Butare, and Icyizere Centre, it was therefore not required of participants to have the ability to read or write. The participants responded to the questionnaire after receiving the required services during their follow-up visit.

### 2.6 Data analysis

The collected data were analyzed using SPSS version 26 software. Descriptive statistics were employed to quantify and summarize data for socio-demographics and knowledge, and bivariate and multivariate analysis were used to assess the factors associated with the use of family planning methods.

### 2.7 Ethical considerations

Before conducting data collection, researchers applied and obtained ethical clearance from IRB/CMHS (Number: 299/CMHS IRB/2022), and then permission to conduct research was requested from Ndera Neuropsychiatric Hospital.

### 2.8 Informed consent, ascent, and incentives

Stabilized women living with mental illness and epilepsy were contacted and explained the study's aim. Before data collection women who were willing to participate in the study signed the consent form. Anonymity was guaranteed by using codes instead of names. Participants were explained that there would be no consequences for participating or refusing to participate in the study and that they could withdraw from the study at any time. The ascent form was also used to ensure that minor participants who met the criteria to participate in the study were still able to participate in the study. In such cases, a legal representative or guardian was approached for their consent on behalf of the participant. This process involved explaining the study's aim and obtaining their permission for the participants to take part. The participants' legal guardians provided written informed consent to participate in this study. They were also informed that the participant's anonymity would be protected and that they could withdraw their consent at any time. By obtaining ascent from both the participant and their representative or guardian, the researchers ensured that the rights and well-being of all participants were respected, even in situations where informed consent may not have been fully possible. There were also incentives for participating in this study. To ensure anonymity, the consent and ascent were kept separate from the questionnaire.

## 3 Results

### 3.1 Socio-demographic and economic characteristics of participants

A total of 289 women of reproductive age participated in the study, making a participation rate of 73%. Tables 1 and 2 provide an overview of the participants' socio-demographic and clinical mental health characteristics. Most participants (63.7%; *n* = 184) were older than 30 years of age. Of all the participants, 12.8% (*n* = 37) were single, while 19.7% (*n* = 57) were legally married and lived with their husbands. Regarding the education level, (3.1%; *n* = 9) of women had no education, while slightly more than half (51.2%; *n* = 148) had a primary level of education. Protestant religious believers (44.3%; *n* = 128) and Catholic religious believers (41.5%; *n* = 120) were predominant among participants in this study. Regarding the socio-economic conditions, the majority of participants (77.5%; *n* = 224) reported that they were unemployed, whereas 39.8% (*n* = 115) were classified in Category 1 and 30.1% (*n* = 87) were in Category 2.

**Table 1:** Demographic characteristics.

Characteristics	Frequency (*n*)	Percentage (%)
Age		
Less than or equal to 20	29	10
21–25	34	11.8
26–30	42	14.5
Greater than 30	184	63.7*
Marital status		
Single, never married	37	12.8
Married legally	57	19.7
Widowed	153	52.9*
Divorced/separated	5	1.7
Not legally married	37	12.8
Religion		
Protestant	128	44.3*
Catholic	120	41.5
Muslim	7	2.4
7th Adventist	32	11.1
No religion	2	0.7
Education level		
No-educated	9	3.1
Primary	148	51.2*
Secondary	106	36.7
University	26	9
No-educated	9	3.1
Ubudehe category		
Category 1	115	39.8*
Category 2	87	30.1
Category 3	81	28
Occupation		
Unemployed	224	77.5*
Part-time job	12	4.2
Student	6	2.1
Housemaid	3	1
Farming/cultivating crops	10	3.5
Self - employed	21	7.3
Government employee	13	4.5

* Indicate the highest percentage.

**Table 2:** Participants clinical mental health characteristics.

Characteristics	Frequency (*n*)	Percentage (%)
Diagnosis		
Schizophrenia	94	31.9^a^
Depression	25	8.5
Bipolar	90	30.5
Brief psychotic disorder	46	15.6
Epilepsy	40	13.6
Time with psychiatric illness in months		
1–24	44	15.2
25–48	43	14.9
49 and above	202	69.9^a^
Duration of treatment and follow-up at a psychiatric clinic		
1–24	54	18.7
25–48	45	15.6
49 and above	190	65.7^a^
Started medication		
Yes	287	99.3^a^
No	2	0.7

^a^ Indicate the highest percentage.

### 3.2 Mental health characteristics of the participants

The majority of respondents (69.9%; *n* = 202) spent above 49 months living with a psychiatric illness and (30.5%; *n* = 90) of the respondents suffered from bipolar while (31.9%) were suffering from schizophrenia and (99.3%; *n* = 287) had already started taking medication (65.7; *n* = 190) had spent more than 49-month taking medication and making follow up at the psychiatric clinic.

### 3.3 Reproductive characteristics of the respondents

Most of the participants (81%; *n* = 234) had sex in life and (59.8%; *n* = 140) had their first sexual intercourse at the age of 18 years and above, of which 63.75% had a consenting first sexual intercourse, participants, while 31.6% had forced sex in their life. A relatively small number (25.6%) had a sexually transmitted illness history. Regarding pregnancy, (63.7%; *n* = 184) did not have a history of pregnancy while (95.7%; *n* = 176) had never given birth. Of those with pregnancy history, (47.1; *n* = 136) had 3 or fewer pregnancies and (46.7%; *n* = 135) had between 1 and 3 biological children. For participants who had fallen pregnant, more than half (53.8%) of the pregnancies were wanted, and 50.5% were planned. The women who had given previous delivery in 1 year or less at (48.8; *n* = 141). Of the participants who gave birth, a significant number (91.4%) gave birth after being diagnosed with a psychiatric disorder (91.4; *n* = 117). Among the participants only (60.9%, *n* = 176) wanted more children in the future (Table 3).

**Table 3:** Reproductive characteristics of the respondents.

Characteristics	Frequency (*n*)	Percentage (%)
Ever had sex		
Yes	234	81^a^
No	55	19
Age you had first sex in years		
Less than 18	94	40.2
18 and above	140	59.8
First sex forced		
Yes	85	36.3
No	149	63.7
Ever had forced sex		
Yes	74	31.6
No	160	68.4
STI history		
Yes	60	25.6
No	174	74.4
Ever pregnant		
Yes	184	63.7
No	105	36.3
Number of pregnancies		
3 or less	136	47.1
Greater than 3	48	16.6
Pregnancies after psychiatric		
1–3	117	91.4^a^
Greater than 3	11	8.6
Last pregnancy wanted		
Yes	99	53.8
No	85	46.2
Last pregnancy planned		
Yes	93	50.5
No	91	49.5
Ever give birth		
Yes	176	95.7^a^
No	8	4.3
Previous delivery		
1 year or less	141	48.8
1–2 years	30	10.4
Above 2 years	5	1.7
Biological children		
1–3	135	46.7
4–6	33	11.4
Above 7	8	2.8
Want more children in the future		
Yes	176	60.9
No	105	36.3
Don't know	8	2.8

^a^ Indicate the highest percentage.

### 3.4 Family planning awareness, use, and self-reported source of information

The majority (96.9%, *n* = 280) of study participants were aware of FP methods, (67.8%, *n* = 196) had used one of the family planning methods in life and only (51.9%, *n* = 150) used the family planning methods at the time of data collection (see Table 4). Those who would like to use a family planning method in the future were at (26%, *n* = 75).

**Table 4:** Family planning awareness and use.

Variable	Frequency (*n*)	Percentage (%)
Do you know methods of family planning		
Yes	280	96.9
No	9	3.1
Source of information		
Healthcare providers	186	28.7
Family members	138	49.3
Friends	121	18.7
Mass media	106	16.4
School	95	14.7
Have you used any family planning methods?		
Yes	196	67.8
No	93	32.2
Currently using family planning method		
Yes	150	51.9
No	46	15.9
Would you like to use a family planning method in the future?		
Yes	75	26
No	52	18
Don't remember	10	3.5
Don't know	2	0.7

The women had information about the methods of family planning from health care providers (28.7%, *n* = 186) from family members, (49.3%, *n* = 138), from friends, (18.7%, *n* = 121), from mass media, (16.4%, *n* = 106), from school (14.7%, *n* = 95).

### 3.5 Family planning awareness and use based on the type of methods

The most commonly known methods were the injectable (17.3%; *n* = 256), oral contraceptive pill (17.3%; *n* = 254), contraceptive implant (15.0%; *n* = 220), rhythm (14.2%; *n* = 208) condoms (13.3%; *n* = 196), and intrauterine device (9.6%; *n* = 141), tube litigation(4.8%; *n* = 71), vasectomy(3.2%; *n* = 47); breastfeeding (2.1%; *n* = 31) and (2.8%; *n* = 41) reported the withdrawal as natural method (Table 5).

**Table 5:** Family planning awareness and use based on the type of methods.

Variables	Family planning method awareness (*N* = 280)		Family planning method utilization (*n* = 196)	
	Yes	Percentage (%)	Yes	Percentage (%)
Family planning methods				
Rhythm	208	14.2	155	15.2
Withdrawal	41	2.8	28	2.7
Breastfeeding	31	2.1	20	2.0
Vasectomy	47	3.2	31	3.0
Tubal ligation	71	4.8	47	4.6
Implants	220	15.0	152	14.9
IUD	141	9.6	99	9.7
Injectables	256	17.4	179	17.5
Pill (OCP)	254	17.3	179	17.5
Condom	196	13.3	131	12.8
No response	4	0.3	2	0.4

Regarding family planning utilization, the most frequently used methods of contraception were pills (17.5%; *n* = 179) and injectables (17.5%; *n* = 179). On the other hand, (14.9%; *n* = 152), (12.8%; *n* = 131), and (9.7%; *n* = 99) of women were using implants, condoms, and intrauterine devices respectively. The participants who used the tube litigation were (4.6%; *n* = 47), vasectomy (3.0%; *n* = 31). Regarding the natural methods breastfeeding was used by (2.0%; *n* = 20) and (2.7%; *n* = 155) reported having used withdrawal as a natural method.

### 3.6 Factors associated with family planning utilization

Table 6 shows the association between different factors with family planning utilization. On multivariable analysis, women who had primary school were more likely to use FP than those who had a high level of education (AOR = 5.6, 95% CI: 2.0, 15.9). Women who were married (AOR = 51.4, 95% CI: 11.9–22) and those who were single (AOR = 66.4, 95% CI: 9.8, 44) were more likely to use FP (AOR = 0.31, 95% CI: 0.19, 0.51) compared to those who were divorced or separated. The women who knew the contraceptive method (AOR = 5.8, 95% CI: 0.6, 49) were more likely to use FP than those who were not aware of the contraceptive. On multivariable analysis, women who suffer from Brief psychotic disorders had six times the odds of using family planning than those who didn't suffer from it (AOR = 2.7, 95% CI: 1.1, 6.6).

**Table 6:** Factors associated with family planning utilization.

Variables	Family planning utilization		AOR (95% CI)	*P*-value	COR (95% CI)	*P*-value
	Yes	No				
Religion						
No religion	1 (50)	1 (50)	1	1	1	
Protestant	86 (67.1)	42 (32.8)	2.0 (0.6–33.5)	0.61	3.1 (0.1–68.8)	0.45
Catholic	87 (72.5)	33 (27.5)	2.6 (0.1–43.3	0.49	5.4 (0.2–118.9)	0.28
Muslim	4 (57.1)	3 (42.8)	1.3 (0.0–31.1)	0.85	1.77 (0.05–58.2)	0.74
7th Adventist	18 (56.2)	14 (43.7)	1.2 (0.0–22.4)	0.86	2.3 (0.09–54.6)	0.59
Education level						
Non-educated	7 (77.7)	2 (22.2)	3.5 (0.7–16.8)	0.11	3.1 (0.4–23.1)	0.26
Primary	111 (75.0)	37 (25.0)	3 (2.0–4.3)	0.00	5.6 (2.0–15.9)	0.00*
Secondary	66 (62.2)	40 (37.7)	1.6 (1.1–2.4)	0.01	2.9 (1.0–8.2)	0.04
University	12 (46.1)	14 (53.8)	1		1	
Marital status						
Single, never married	32 (86.4)	5 (13.5)	5.8 (2.1–15.7)	0.00	66.4 (9.8–44)	0.00*
Married legally	51 (89.4)	6 (10.5)	7.7 (3.1–19.1)	0.00	51.4 (11.9–22)	0.00*
Divorced/separated	2 (40)	3 (60)	0.6 (0.0–3.7)	0.59	0.2 (0.03–1.58)	0.13
Non-married partner/	31 (83.7)	6 (16.2)	4.7 (1.8–11.9)	0.00	1	
Widowed	80 (52.2)	73 (47.7)	1		1	
Ubudehe						
Category 1	84 (73.0)	31 (26.9)	1		1	
Category 2	58 (66.6)	29 (33.3)	0.7 (0.4–1.3)	0.32	0.6 (0.2–1.4)	0.27
Category 3	51 (62.9)	30 (37.0)	0.6 (0.3–1.1)	0.13	0.7 (0.2–2.4)	0.62
No category	3 (50.0)	3 (50.0)	0.3 (0.0–1.9)	0.23	1	
Number of children						
Less than 3	116 (85.9)	19 (14.0)	0.87 (0.1–7.4)	0.90	0.6 (0.06–6.4)	0.68
4–6	32 (96.9)	1 (3.0)	4.5 (0.25–82)	0.30	2.6 (0.1–5.5)	0.53
7 and above	7 (87.5)	1 (12.5)	1		1	
Ever had forced sex?						
Yes	61 (82.4)	13 (17.5)	1.4 (0.7–2.9)	0.28	0.97 (0.3–2.6)	0.96
No	122 (76.2)	38 (23.7)	1		1	
Unwanted pregnancy						
Yes	95 (91.3)	9 (9.6)	8.7 (4.1–18.4)	0.00	1.6 (0.6–4.4)	0.30
No	101 (54.5)	84 (45.4)	1			
Know contraception method						
Yes	194 (69.2)	86 (30.7)	7.8 (1.6–38.7)	0.01	5.8 (0.6–49)	0.038*
No	2 (22.2)	7 (77.7)	1			
Want more children in the future						
Yes	105 (59.6)	71 (40.3)	1		1	
No	86 (81.9)	19 (18.1)	3.0 (1.7–5.4)	0.00	1.4 (0.5–3.5)	0.4
Don't know	5 (62.5)	3 (37.5)	1.1 (0.2–4.8)	0.8	0.8 (0.0–7.8)	0.8
Ever had sex						
Yes	183 (78.2)	51 (21.7)	11.5 (5.7–23.2)	0.00	1	
No	13 (23.6)	42 (76.3)	1		1	
Ever given birth						
Yes	155 (88.0)	21 (11.9)	2.4 (0.4–12.9)	0.2	2 (0.3–11.8)	0.3
No	6 (75.0)	2 (25.0)	1			
Time with mental illness						
0–6 months	4 (80.0)	1 (20.0)	1.6 (0.3–8.2)	0.5	1	
7–11 months	11 (61.1)	7 (38.8)	0.9 (0.3–2.3)	0.5	1.1 (0.05–22.6)	0.9
12 months and above	181 (68.0)	85 (31.9)	1		1	
Duration of treatment						
0–6 months	7 (77.7)	2 (22.2)	1.8 (0.2–17.0)	0.5	1	
7–11 months	16 (66.6)	8 (33.3)	0.7 (0.2–1.9)	0.4	1	
12 months and above	173 (67.5)	83 (32.4)	1			
Types of diagnosis						
Schizophrenia						
Yes	65 (69.1)	29 (30.8)	1.1 (0.6–1.8)	0.7	1.8 (0.9–3.6)	0.8
No	130 (67.0)	64 (32.9)	1			
Depression						
Yes	20 (80.0)	5 (20.0)	2.0 (0.7–5.5)	0.1	2.8 (0.9–8.3)	0.6
No	175 (66.5)	88 (33.4)	1			
Bipolar						
Yes	60 (66.6)	30 (33.3)	0.9 (0.5–1.5)	0.7	1.5 (0.8–3.1)	0.1
No	135 (68.1)	63 (31.8)	1			
Brief psychotic disorder						
Yes	36 (78.2)	10 (21.7)	1.8 (0.8–3.9)	0.0	2.7 (1.1–6.6)	0.0
No	159 (65.7)	83 (34.3)	1			
Types of diagnosis						
Schizophrenia (Yes)	65 (69.1)	29 (30.8)	1.1 (0.6–1.8)	0.7	1.0 (0.4–2.5)	0.9
Depression (Yes)	20 (80.0)	5 (20.0)	2.0 (0.7–5.5)	0.1	1.8 (0.5–5.9)	0.2
Bipolar (Yes)	60 (66.6)	30 (33.3)	0.9 (0.5–1.5)	0.7	0.8 (0.3–2.2)	0.8
Brief psychotic disorder (Yes)	36 (78.2)	10 (21.7)	Omitted			
Epileptic (Yes)	19 (47.5)	21 (52.5)	0.08 (0.02–0.3)	0.00	0.3 (0.1–1.0)	0.3

* Indicate the highest percentage.

## 4 Discussion

This cross-sectional study attempted to assess knowledge, utilization of FP, and associated factors among women with a mental illness and epilepsy history in Rwanda. It included 289 women who were scheduled to be discharged from a psychiatric hospital and those who visited the outpatient services of the same hospital and its affiliated branches from October 2022 to February 2023. The majority of respondents have been living with a psychiatric condition for more than 49 months, and 31.9% were diagnosed with schizophrenia, while 13.6% had epilepsy. Of the respondents, a significant number had their pregnancies following their psychiatric diagnosis, and more than half (60.9%) had plans for future pregnancies. These findings underscore the FP needs of women with mental illness and epilepsy and reiterate the need to understand the FP use patterns in this vulnerable group.

Contrary to previous studies exploring FP among women with a psychiatric history within Sub-Saharan Africa, which found that this population was less aware of FP than the general population (2, 3), a huge majority of respondents (96.9%) from our study had an increased awareness of FP methods, 67.8% had used at least one FP method in the past, and more than half were using FP. The recent Rwanda Demographic Health survey found lower but closer estimates of the use of FP among married women (64%), while another Rwandan study revealed that 90% of the general population women were aware of family planning (16). Such an unexpected finding lends evidence to the positive uptake of FP among women with mental illness and epilepsy to the same level as women without a mental illness history in Rwanda.

Different factors are likely contributing to this reasonable knowledge of FP. First, Rwanda's efforts to strengthen the health system and to prevent unintended pregnancies, including increasing access to FP services and information (17), might have led to a massive reach of the majority of women of reproductive age, including those with mental health problems. Second, and as evidenced by the findings from our study, healthcare providers are the main source of information on FP for most respondents. Third, the integration of mental health services into primary care might have allowed women with mental health problems who attend these integrated services during their follow-up treatment to benefit from other health-related information, such as FP. It is therefore important that these strategies be maintained to continue accessing various categories of women.

Similar utilization patterns were found in terms of types of FP methods mostly used by women with a psychiatric history compared to the general population (18). In this study, the most frequently used methods of contraception were pills (17.5%) and injectables (17.5%). On the other hand, 14.9%, 12.8%, and 9.7% of women were using implants, condoms, and intrauterine devices respectively. A relatively small proportion of participants who used the tube litigation were (4.6%; *n* = 47), vasectomy (3.0%; *n* = 31). This confirmed the research conducted in Rwanda among women of reproductive age in the general population where pills and injections were the methods most commonly used (16), as well as research conducted in other African countries such as Ethiopia (19), and Uganda (18).

A possible explanation for the low awareness of long-acting reversible methods, intrauterine devices, tube litigation, and vasectomy found in the current study might be that due to the limited availability of these FP methods in resources-limited countries, health information for FP in these settings probably focuses mainly on the most available and affordable methods such as pills and injectable. However, even the natural traditional family planning methods that are less costly are also less known by women in our study, as only a relatively small number of respondents (2.1% and 2.8%) respectively mentioned breastfeeding and withdrawal among known FP methods, a finding that is similar to what was found among reproductive age women in Ethiopia (20). Health systems in low-resource countries should diversify the health information regarding FP to provide reproductive-age women with a wide range of FP methods, including natural FP methods. Particularly women with mental illness and epilepsy have an increased likelihood of inconsistent use, inadequate collaboration, and poor hygiene, which may make it impossible to use an intrauterine device (2); and are likely to discontinue their regular use of FP due to diminished judgment and have a high risk of drug interactions between antipsychotic and oral contraceptives (21). Conversely, injectables can be utilized when compliance cannot be ensured, the risk of discontinuation is high, and there are no contraindications (22). Hence, increased awareness of other FP options might be a benefit to women with mental illness and epilepsy.

Findings from multivariate regression analysis revealed that FP awareness was a predictor of FP use since women with mental illness and epilepsy in the current study who were aware of the contraceptive technique (AOR = 5.8, 95% CI: 0.6, 49) were more likely to use FP than those who were not. Therefore, efforts to increase awareness will eventually increase the amount of use since the gap between knowledge and use of FP is a measure of FP's unmet needs (23). Despite an increased awareness of FP, only 67.8% of the women who took part in our survey reported using any FP methods. This gap between knowledge and use from our study indicates that additional efforts will need to be undertaken to raise awareness of FP among women with mental disorders, to narrow that utilization gap, and to equip them with the necessary information to make FP choices that meet their specific needs.

The stigma, ignorance of the interactions between psychiatric medications and contraceptives, and other illness-related difficulties may have contributed to the differences between awareness and use among our study participants and the general population. The other factor might be because women with severe mental illness engage less with general health services and frequently with mental health professionals, who might not include FP information in their interactions with patients. Exploring these various potential barriers to FP use will require further research.

The multivariate regression analysis also revealed that women with mental illness who only completed primary school were more likely to use FP than those who completed high school (AOR = 5.6, 95% CI: 2.0, 15.9). This is another striking finding from our study since it does not seem to be the case in other studies conducted within Sub-Saharan Africa. Several other studies have shown that lower educational attainment is associated with less FP awareness and use (24), lending more credence to the idea that a lack of education prevents people from changing their contraceptive behavior (25). There are several possible explanations for this different finding. This may indicate that factors that affect FP utilization in such a vulnerable population as women with mental illness could be different from the general population. It can also be explained by the fact that in many cases involving women with mental health; FP decisions might be made by significant others, such as guardians, who might have a higher education level than the women using FP. Also, the study revealed that Women with no education or only primary education are more inclined to participate in high-risk fertility behaviors compared to those with higher levels of education. Each additional year of schooling decreases the likelihood of engaging in these high-risk behaviors by 6%, which may expose them to use FP methods (26).

However, we did not evaluate these factors or the severity of the mental disorder in this investigation and future studies may help enlighten this.

## 5 Limitations

This study does have some limitations. It could be challenging to determine a cause-and-effect link between the variables because the study is cross-sectional. Given the time and funding limitations, this study was unable to expand the sample to include other categories of women attending other settings. The results may not be generalizable to women with mental illness who do not attend psychiatric facilities because the study was carried out in hospitals where patients were believed to have better access to information.

## 6 Conclusion

Despite its limitations, this research is the first to have examined FP awareness and use among Rwandan women with mental illness and epilepsy. Findings revealed that women with mental illness are empowered with the necessary knowledge of FP to the same level as women without a mental illness history, but a significant gap between awareness and use remains. A personalized and systematic approach to empowering women with mental illness on FP methods is vital to meet the FP needs of this vulnerable group. Mental health services should integrate FP information into their routine practices to mitigate the remaining gap. Other FP types such as condoms should be advised to women with mental illness who are of reproductive age regardless of the method utilized due to the protection provided against sexually transmitted diseases. To gain a deeper understanding of the enablers and barriers to accessing family planning among women with mental illness and epilepsy, qualitative studies are needed. Additionally, a study on the interactions between FP contraceptives and psychiatric drugs is required.

## Data Availability

The original contributions presented in the study are included in the article/Supplementary Material, further inquiries can be directed to the corresponding author.
