# Supplementary material for: Family planning knowledge, use, and associated factors among women with mental illness and epilepsy in Rwanda: a cross-sectional study
Source: Front Glob Womens Health. 2024 Oct 25;5:1373051. doi: 10.3389/fgwh.2024.1373051 (PMC11543584; doi:10.3389/fgwh.2024.1373051)
Supplement: Supplementary file 1 [file Table1.docx]

**4.1. Socio-demographics and economic characteristics of participants**

A total of 289 women of reproductive age participated in the study making a participation rate of 73%. Tables 1 & 2 overview the participants’ socio-demographic and clinical mental health characteristics. Most participants (63.7%; n =184) were greater than 30 years. Almost (12.8%; n =37) were single and (19.7%; n =57) were legally married and lived with their husbands. Regarding the education level, (3.1%; n=9) of women were illiterate and (51.2 %; n =148) of them had only primary-level education. The majority of respondents were Catholics (41.5%; n =120) and protestants (44.3%; n =128). Regarding the socio-economic conditions majority of women were in Category 1 (39.8 % n =115) and Category 2 (30.1% n =87) and (77.5%; n =224) of them were unemployed.

**Table 1: Demographic Characteristics**

| **Characteristics** | | | **Frequency**  **(n)** | | | **Percentage**  **(%)** | | | |
| --- | --- | --- | --- | --- | --- | --- | --- | --- | --- |
| **Age** | | |  | | |  | | |  |
| Less than or equal 20 | | | 29 | | | 10 | |  |  |
| 21 - 25 | | | 34 | | | 11.8 | |  |  |
| 26 - 30 | | | 42 | | | 14.5 | |  |  |
| Greater than 30 | | | 184 | | | 63.7* | |  |  |
| **Marital status** | | |  | | |  | | | |
| Single, never married | | | 37 | | | 12.8 | |  |  |
| Married Legally | | | 57 | | | 19.7 | |  |  |
| Widowed | | | 153 | | | 52.9* | |  |  |
| Divorced/separated | | | 5 | | | 1.7 | |  |  |
| Not legally married | | | 37 | | | 12.8 | |  |  |
| **Religion** | | |  | | |  | | | |
| Protestant | | | 128 | | | 44.3* | |  |  |
| Catholic | | | 120 | | | 41.5 | |  |  |
| Muslim | | | 7 | | | 2.4 | |  |  |
| 7th Adventist | | | 32 | | | 11.1 | |  |  |
| No religion | | | 2 | | | 0.7 | |  |  |
| **Education level** | | |  | | |  | | | |
| No-educated | | | 9 | | | 3.1 | |  |  |
| Primary | | | 148 | | | 51.2* | |  |  |
| Secondary | | | 106 | | | 36.7 | |  |  |
| University | | | 26 | | | 9 | |  |  |
| No-educated | | | 9 | | | 3.1 | |  |  |
| **Ubudehe category** | | |  | | |  | | | |
| Category 1 | | | 115 | | | 39.8* | |  |  |
| Category 2 | | | 87 | | | 30.1 | |  |  |
| Category 3 | | | 81 | | | 28 | |  |  |
|  | | |  | | |  | |  |  |
| **Occupation** | | |  | | |  | | |  |
| Unemployed | | | 224 | | | 77.5* | |  |  |
| Part time job | | | 12 | | | 4.2 | |  |  |
| Student | | | 6 | | | 2.1 | |  |  |
| House maid | | | 3 | | | 1 | |  |  |
| Farming/cultivating crops | | | 10 | | | 3.5 | |  |  |
| Self - employed | | | 21 | | | 7.3 | |  |  |
| Government employee | | | 13 | | | 4.5 | |  |  |

## 4.2. Clinical mental health characteristics of the participants

The majority of respondents (69.9%; n= 202) spent above 49 months living with the psychiatric illness and (30.5%; n=90) of the respondents suffered from bipolar while (31.9%) were suffering from schizophrenia and ( 99.3%; n= 287) had already starting taking medication ( 65.7; n= 190) had spent more than 49 month taking medication and making follow up at psychiatric clinic.

**Table 3: Participants Clinical mental health characteristics**

| **Characteristics** | | | **Frequency**  **(n)** | | | **Percentage**  **(%)** | | |  |
| --- | --- | --- | --- | --- | --- | --- | --- | --- | --- |
| **Diagnosis** | | |  | | |  | | |  |
| Schizophrenia | | | 94 | | | 31.9* | | | |
| Depression | | | 25 | | | 8.5 | | | |
| Bipolar | | | 90 | | | 30.5 | | | |
| Brief psychotic disorder | | | 46 | | | 15.6 | | | |
| Epilepsy | | | 40 | | | 13.6 | | | |
| **Time with psychiatric illness in months** | | | | | | | | |  |
| 1- 24 | | | 44 | | | 15.2 | | | |
| 25 - 48 | | | 43 | | | 14.9 | | | |
| 49 and above | | | 202 | | | 69.9* | | | |
| **Duration on treatment and follow up at psychiatric clinic** | | | | | | | | |  |
| 1- 24 | | | 54 | | | 18.7 | | | |
| 25 - 48 | | | 45 | | | 15.6 | | | |
| 49 and above | | | 190 | | | 65.7* | | | |
| **Started medication** | | |  | | |  | | |  |
| Yes | | | 287 | | | 99.3* | | | |
| No | | | 2 | | | 0.7 | | |  |

*: Indicate the highest percentage

## 4.3. Reproductive Characteristics of the respondents

The majority of the participants (81%; n=234) had sex in life and (59.8%; n=140) had first sexual intercourse in 18 years and above. For (63.75; n=149) of women, the first sex they had were not forced. Only (31.6%; n=74) had had forced sex in their life and (25.6%; n=60) had STI history. Regarding the pregnancy, (63.7 %; n=184 didn’t have an history of pregnancy while (95.7%; n=176) had ever give birth. Of these with pregnancy history, (47.1; N=136) had 3 or less pregnancies and (46.7%; n=135) had between 1-3 biological children. The women who reported that the last pregnancy was wanted were (53.8, n=99) while for these the pregnancy was planned were (50.5%; n=93). The women who had give Previous delivery in 1year or less at (48.8; n= 141). The women had 3 or less pregnancies after psychiatric having disorder were at 91.4; n=117). Among the participants only (60.9%, n= 176) wanted more children in the future. (Table 3).

| **Characteristics** | | **Frequency**  **(n)** | | **Percentage**  **(%)** | |
| --- | --- | --- | --- | --- | --- |
| **Ever had sex** | |  | |  | |
| Yes | | 234 | | 81 | |
| No | | 55 | | 19 | |
| **Age you had first sex in years** | | | | | |
| Less than 18 | | 94 | | 40.2 | |
| 18 and above | | 140 | | 59.8 | |
| **First sex forced** | |  | |  | |
| Yes | | 85 | | 36.3 | |
| No | | 149 | | 63.7 | |
| **Ever had forced sex** | |  | |  | |
| Yes | | 74 | | 31.6 | |
| No | | 160 | | 68.4 | |
| **STI history** | |  | |  | |
| Yes | | 60 | | 25.6 | |
| No | | 174 | | 74.4 | |
| **Ever pregnant** | |  | |  | |
| Yes | | 184 | | 63.7 | |
| No | | 105 | | 36.3 | |
| **Number of pregnancies** | |  | |  | |
| 3 or less | | 136 | | 47.1 | |
| Greater than 3 | | 48 | | 16.6 | |
| **Pregnancies after psychiatric** | | | | | |
| 1-3 | | 117 | | 91.4 | |
| Greater than 3 | | 11 | | 8.6 | |
| **Last pregnancy wanted** | |  | |  | |
| Yes | | 99 | | 53.8 | |
| No | | 85 | | 46.2 | |
| **Last pregnancy planned** | |  | |  | |
| Yes | | 93 | | 50.5 | |
| No | | 91 | | 49.5 | |
| **Ever give birth** | |  | |  | |
| Yes | | 176 | | 95.7 | |
| No | | 8 | | 4.3 | |
| **Previous delivery** | |  | |  | |
| 1 year or less | | 141 | | 48.8 | |
| 1 – 2 years | | 30 | | 10.4 | |
| Above 2 years | | 5 | | 1.7 | |
| **Biological children** | |  | |  | |
| 1-3 | | 135 | | 46.7 | |
| 4-6 | | 33 | | 11.4 | |
| Above 7 | | 8 | | 2.8 | |
| **Want more children in the future** | | | | | |
| Yes | | 176 | | 60.9 | |
| No | | 105 | | 36.3 | |
| Don’t know | | 8 | | 2.8 | |

*: Indicate the highest percentage

## 4.4. Family planning awareness, use, and self-reported source of information

The majority (96.9%, n=280) of study participants were aware of FP methods, (67.8%, n=196) had used one of the family planning methods in life and only (51.9%, n=150) used the family planning methods at the time of data collection (Table 4). Those who would like to use a family planning method in the future were at (26%, n=75).

The women had information about the methods of family planning from health care providers (28.7%, n=186) from family members, (49.3%, n=138). from friends, (18.7%, n=121), from mass media, (16.4%, n=106), from school (14.7%, n=95)

| **Variable** | | **Frequency**  **(n)** | | | **Percentage**  **(%)** | |  |
| --- | --- | --- | --- | --- | --- | --- | --- |
|  | |  | | |  | |  |
| **Do you know methods of family planning** | | | | | | | |
| **Yes** | | | | 280 | | | 96.9 |
| **No** | | | | 9 | | | 3.1 |
| **Source of information** | | | |  | | |  |
| Healthcare providers, | | | | 186 | | | 28.7 |
| Family members, | | | | 138 | | | 49.3 |
| Friends, | | | | 121 | | | 18.7 |
| Mass media | | | | 106 | | | 16.4 |
| School | | | | 95 | | | 14.7 |
| **Have you used any family planning methods?** | | | | | | | |
| Yes | | | | 196 | | | 67.8 |
| No | | | | 93 | | | 32.2 |
| **Currently using family planning method** | | | | | | | |
| Yes | | | | 150 | | | 51.9 |
| No | | | | 46 | | | 15.9 |
| **Would you like to use a family planning method in the future** | | | | | | | |
| Yes | | | | 75 | | | 26 |
| No | | | | 52 | | | 18 |
| Don’t remember | | | | 10 | | | 3.5 |
| Don’t know | | | | 2 | | | 0.7 |
|  | |  | | |  | | |

**4.5 Family planning awareness and use based on the type of methods**

The most commonly known methods were the injectable (17.3%; n =256), oral contraceptive pill (17.3%; n =254) , contraceptive implant (15,0%; n =220 ), rhythm (14,2%; n = 208) condoms (13.3%; n =196 ), and intrauterine device (9.6%; n = 141), tube litigation(4.8%; n=71), vasectomy(3.2%; n = 47); breastfeeding (2,1%; n =31 ) and (2.8%; n = 41) reported the withdrawal as natural method ( Table 5).

Regarding family planning utilization, the most frequently used method of contraception were pills (17.5%; n =179) and injectables (17.5%; n = 179). On the other hand, (14.9%; n = 152), (12.8%; n = 131), and (9.7%; n = 99) of women were using implant, condoms, and intrauterine devices respectively. The participants who used the tube litigation were (4.6%; n=47), vasectomy (3.0%; n = 31). Regarding the natural methods breastfeeding were used by (2,0%; n =20) and (2.7%; n = 155) reported having used the withdrawal as natural method.

| **Variables** | **Family planning**  **Method Awareness (N=280)** | | **Family planning method utilization (n= 196)** | | | | |
| --- | --- | --- | --- | --- | --- | --- | --- |
|  | **Yes** | **Percentage (%)** | **Yes** |  | **Percentage (%)** | |  |
| **Family planning methods** | | | | | | | |
| Rhythm | 208 | 14.2 | 155 | | | 15.2 | |
| Withdrawal | 41 | 2.8 | 28 | | | 2.7 | |
| Breastfeeding | 31 | 2.1 | 20 | | | 2.0 | |
| Vasectomy | 47 | 3.2 | 31 | | | 3.0 | |
| Tubal ligation | 71 | 4.8 | 47 | | | 4.6 | |
| Implants | 220 | 15.0 | 152 | | | 14.9 | |
| IUD | 141 | 9.6 | 99 | | | 9.7 | |
| Injectables | 256 | 17.4 | 179 | | | 17.5 | |
| Pill (OCP) | 254 | 17.3 | 179 | | | 17.5 | |
| Condom | 196 | 13.3 | 131 | | | 12.8 | |
| **No response** | 4 | 0.3 | 2 | | | 0.4 | |

## 4.6. Factors associated with family planning utilization.

Table 6 shows the association between different factors in family planning utilization

On multivariable analysis, women who had primary school were more likely to use FP than those who had a high level of education (AOR= 5.6,95% CI: 2.0, 15.9). Women who were married (AOR= 51.4,95% CI: 11.9-22) and those who were single (AOR = 66,4, 95% CI: 9.8, 44) were more likely to use FP (AOR = 0 .31, 95% CI: 0.19, 0.51) compare to those who were divorced or separated. The women who knew the contraceptive method (AOR= 5.8,95% CI: 0.6, 49) were more likely to use FP than those who were not aware of the contraceptive. On multivariable analysis, women who suffer from Brief psychotic disorders had six times the odds of using family planning than those who didn’t suffer from it (AOR = 2.7, 95% CI: 1.1, 6.6).

| **Variables** | | | **Family planning utilization** | | | | **AOR (95%CI)** | | **P-value** | **COR (95%CI)** | **P-value** |
| --- | --- | --- | --- | --- | --- | --- | --- | --- | --- | --- | --- |
|  | | | **Yes** | | **No** | |  | |  |  |  |
| **Religion** | | |  | |  | |  | |  |  |  |
| No religion | | | 1(50) | | 1(50) | | 1 | | 1 | 1 |  |
| Protestant | | | 86(67.1) | | 42(32.8) | | 2.0 (0.6-33.5) | | 0.61 | 3.1(0.1-68.8) | 0.45 |
| Catholic | | | 87(72.5) | | 33(27.5) | | 2.6 (0.1-43.3 | | 0.49 | 5.4(0.2-118.9) | 0.28 |
| Muslim | | | 4(57.1) | | 3(42.8) | | 1.3 (0.0-31.1) | | 0.85 | 1.77(0.05-58.2) | 0.74 |
| 7^th^ Adventist | | | 18(56.2) | | 14(43.7) | | 1.2 (0.0-22.4) | | 0.86 | 2.3(0.09-54.6) | 0.59 |
| **Education level** | | |  | |  | |  | |  |  |  |
| Non-educated | | | 7(77.7) | | 2(22.2) | | 3.5(0.7-16.8) | | 0.11 | 3.1(0.4-23.1) | 0.26 |
| Primary | | | 111(75.0) | | 37(25.0) | | 3(2.0-4.3) | | 0.00 | 5.6(2.0-15.9) | 0.00* |
| Secondary | | | 66(62.2) | | 40(37.7) | | 1.6 (1.1-2.4) | | 0.01 | 2.9(1.0-8.2) | 0.04 |
| University | | | 12(46.1) | | 14(53.8) | | 1 | |  | 1 |  |
| **Marital status** | | |  | |  | |  | |  |  |  |
| Single, never married | | | 32(86.4) | | 5(13.5) | | 5.8(2.1-15.7) | | 0.00 | 66.4(9.8-44) | 0.00* |
| Married legally | | | 51(89.4) | | 6(10.5) | | 7.7(3.1-19.1) | | 0.00 | 51.4(11.9-22) | 0.00* |
| Divorced/separated | | | 2(40) | | 3(60) | | 0.6(0.0-3.7) | | 0.59 | 0.2(0.03-1.58) | 0.13 |
| Non-married partner/ | | | 31(83.7) | | 6(16.2) | | 4.7(1.8-11.9) | | 0.00 | 1 |  |
| Widowed | | | 80(52.2) | | 73(47.7) | | 1 | |  | 1 |  |
| **Ubudehe** | | |  | |  | |  | |  |  |  |
| Category 1 | | | 84(73.0) | | 31(26.9) | | 1 | |  | 1 |  |
| Category 2 | | | 58(66.6) | | 29(33.3) | | 0.7(0.4-1.3) | | 0.32 | 0.6 (0.2-1.4) | 0.27 |
| Category 3 | | | 51(62.9) | | 30(37.0) | | 0.6(0.3-1.1) | | 0.13 | 0.7(0.2-2.4) | 0.62 |
| No category | | | 3(50.0) | | 3(50.0) | | 0.3(0.0-1.9) | | 0.23 | 1 |  |
| **Number of children** | | | | | | | | | | | |
| Less than 3 | | | 116(85.9) | | 19(14.0) | | 0.87 (0.1-7.4) | | 0.90 | 0.6 (0.06-6.4) | 0.68 |
| 4-6 | | | 32(96.9) | | 1(3.0) | | 4.5(0.25-82) | | 0.30 | 2.6(0.1-5.5) | 0.53 |
| 7 and above | | | 7(87.5) | | 1(12.5) | | 1 | |  | 1 |  |
| **Ever had forced** sex | | |  | |  | |  | |  |  |  |
| Yes | | | 61(82.4) | | 13(17.5) | | 1.4 (0.7-2.9) | | 0.28 | 0.97(0.3-2.6) | 0.96 |
| No | | | 122(76.2) | | 38(23.7) | | 1 | |  | 1 |  |
| **Unwanted Pregnancy** | | | | | | | | | | | |
| Yes | | | 95(91.3) | | 9 (9.6) | | 8.7(4.1-18.4) | | 0.00 | 1.6(0.6-4.4) | 0.30 |
| No | | | 101(54.5) | | 84(45.4) | | 1 | |  |  |  |
| **Know contraception method** | | | | | | | | | | | |
| Yes | | | 194(69.2) | | 86(30.7) | | 7.8(1.6-38.7) | | 0.01 | 5.8(0.6-49) | 0.038* |
| No | | | 2(22.2) | | 7(77.7) | | 1 | |  |  |  |
| **Want more children in the future** | | |  | |  | |  | |  |  |  |
| Yes | | | 105(59.6) | | 71(40.3) | | 1 | |  | 1 |  |
| No | | | 86(81.9) | | 19(18.1) | | 3.0(1.7-5.4) | | 0.00 | 1.4(0.5-3.5) | 0.4 |
| Don’t know | | | 5(62.5) | | 3(37.5) | | 1.1(0.2-4.8) | | 0.8 | 0.8(0.0-7.8) | 0.8 |
| **Ever had sex** | | |  | |  | |  | |  |  |  |
| Yes | | | 183(78.2) | | 51(21.7) | | 11.5(5.7-23.2) | | 0.00 | 1 |  |
| No | | | 13(23.6) | | 42(76.3) | | 1 | |  | 1 |  |
| **Ever give birth** | | |  | |  | |  | |  |  |  |
| Yes | | | 155(88.0) | | 21(11.9) | | 2.4(0.4-12.9) | | 0.2 | 2(0.3-11.8) | 0.3 |
| No | | | 6(75.0) | | 2(25.0) | | 1 | |  |  |  |
| **Time with mental illness** | | |  | |  | |  | |  |  |  |
| 0-6 months | | | 4(80.0) | | 1(20.0) | | 1.6(0.3-8.2) | | 0.5 | 1 |  |
| 7-11 months | | | 11(61.1) | | 7(38.8) | | 0.9(0.3-2.3) | | 0.5 | 1.1(0.05-22.6) | 0.9 |
| 12 months and above | | | 181(68.0) | | 85(31.9) | | 1 | |  | 1 |  |
| **Duration of treatment** | | |  | |  | |  | |  |  |  |
| 0-6 months | | | 7(77.7) | | 2(22.2) | | 1.8(0.2-17.0) | | 0.5 | 1 |  |
| 7-11 months | | | 16(66.6) | | 8(33.3) | | 0.7(0.2-1.9) | | 0.4 | 1 |  |
| 12 months and above | | | 173(67.5) | | 83(32.4) | | 1 | |  |  |  |
| **Types of Diagnosis** | | |  | |  | |  | |  |  |  |
| Schizophrenia | | |  | |  | |  | |  |  |  |
| Yes | | | 65(69.1) | | 29(30.8) | | 1.1(0.6-1.8) | | 0.7 | 1.8(0.9-3.6) | 0.8 |
| No | | | 130(67.0) | | 64(32.9) | | 1 | |  |  |  |
| Depression | | |  | |  | |  | |  |  |  |
| Yes | | | 20(80.0) | | 5(20.0) | | 2.0 (0.7-5.5) | | 0.1 | 2.8(0.9-8.3) | 0.6 |
| No | | | 175(66.5) | | 88(33.4) | | 1 | |  |  |  |
| Bipolar | | |  | |  | |  | |  |  |  |
| Yes | | | 60(66.6) | | 30(33.3) | | 0.9(0.5-1.5) | | 0.7 | 1.5(0.8-3.1) | 0.1 |
| No | | | 135(68.1) | | 63(31.8) | | 1 | |  |  |  |
| Brief psychotic disorder | | |  | |  | |  | |  |  |  |
| Yes | | | 36(78.2) | | 10(21.7) | | 1.8(0.8-3.9) | | 0.0 | 2.7(1.1-6.6) | 0.0 |
| No | | | 159(65.7) | | 83(34.3) | | 1 | |  |  |  |
| **Types of Diagnosis** | | |  | |  | |  | |  |  |  |
| Schizophrenia (Yes) | | | 65(69.1) | | 29(30.8) | | 1.1(0.6-1.8) | | 0.7 | 1.0(0.4-2.5) | 0.9 |
| Depression ( Yes) | | | 20(80.0) | | 5(20.0) | | 2.0(0.7-5.5) | | 0.1 | 1.8(0.5-5.9) | 0.2 |
| Bipolar (Yes) | | | 60(66.6) | | 30(33.3) | | 0.9(0.5-1.5) | | 0.7 | 0.8(0.3-2.2) | 0.8 |
| Brief psychotic disorder (Yes) | | | 36(78.2) | | 10(21.7) | | Omitted | |  |  |  |
| Epileptic (Yes) | | | 19(47.5) | | 21(52.5) | | 0.08(0.02-0.3) | | 0.00 | 0.3(0.1-1.0) | 0.3 |
|  |  |  | |  | |  | |  |  |  |  |
